# Cancer subtypes classification using long non-coding RNA

**DOI:** 10.18632/oncotarget.10213

**Published:** 2016-06-21

**Authors:** Ronan Flippot, Gabriel G. Malouf, Xiaoping Su, Roger Mouawad, Jean-Philippe Spano, David Khayat

**Affiliations:** ^1^ Groupe Hospitalier Pitié-Salpêtrière, Department of Medical Oncology, University Pierre and Marie Curie (Paris VI), Institut Universitaire de Cancérologie, AP-HP, Paris, France; ^2^ Department of Bioinformatics and Computational Biology, University of Texas MD Anderson Cancer Center, Houston, TX, USA

**Keywords:** long non-coding RNAs, lncRNAs, cancer, classification, prognosis

## Abstract

Inter-tumor heterogeneity might explain divergent clinical evolution of cancers bearing similar pathological features. In the last decade, genomic has highly improved tumor subtypes classification through the identification of oncogenic or tumor suppressor drivers. In addition, epigenetics and long non-coding RNAs (lncRNAs) are emerging as new fields for investigation, which might also account for tumor heterogeneity. There is growing evidence that modifications of lncRNA expression profiles are involved in cancer progression through epigenetic regulation, activation of pro-oncogenic pathways and crosstalks with other RNA subtypes. Consequently, the study of lncRNA expression profile will be a key factor in the future for charting cancer subtype classifications as well as defining prognostic and progression biomarkers. Herein we discuss the interest of lncRNA as potent prognostic and predictive biomarkers, and provide a glimpse on the impact of emerging cancer subtypes classification based on lncRNAs.

## INTRODUCTION

The attempts to classify cancer subtypes answer the need for better patients' management. Indeed, the identification of different prognostic groups, predictive factors of response to therapy, or specific oncogenic mechanisms help the clinician to better monitor patients, to personalize treatments at an individual scale, and to avoid the harm caused by a useless regimen. These classifications were historically based on clinical, pathological, and laboratory markers. Among them, the IMDC (international metastatic renal cell carcinoma database) prognostic classification for renal cell carcinoma, based on clinical and laboratory markers, defines the prognosis and therapeutic options for patients in the first-line setting [1]; the pathological subtyping of breast cancer identifies the patients that will mostly benefit from endocrine or anti-HER2 therapy [2]; the identification of deficient mismatch repair in colon cancer categorizes a subset of patients that will less likely benefit from adjuvant chemotherapy [3].

In the last decade, predictive markers of tumor recurrence have benefited from the increasing accessibility of molecular biology and the arrival of next-generation sequencing technologies, that allow for more accurate depiction of genomic and epigenetic alterations in cancer. In breast cancer, the transcriptomic profiling of 50 genes has identified four distinct subtypes of breast cancer with specific ontogeny [4], and a recurrence score based on the expression of 21 genes has been validated to determine the patients who will benefit from adjuvant treatment [5]. Another example is the CpG island methylator phenotype (CIMP), initially discovered in colon cancer that has since been reported to be associated with *IDH1* and *IDH2* mutations in glioblastoma [6] and associated with improved prognosis. The latter association is explained by epigenetic reprogramming mediated by *IDH1* and *IDH2* alterations [7] which are putative targets of experimental treatments [8]. Thus, it is impressive to see that molecular medicine has entered clinical practice and led to a better tumor subtype characterization, with new emphasis on the interplay between genetic and epigenetic aberrations in solid tumors, and new developments in targeted therapies.

However, despite these advances, many questions remain unsolved. Indeed, primary resistance to targeted therapies might occur despite of the presence of a targetable alteration: response rates reach a ceiling at 65% in *BRAF*-mutated melanoma treated by *BRAF* inhibitors [9], and 71% in *EGFR*-mutated lung cancer [10]. In addition, similar genetic alterations in different cancers are not always associated with identical outcomes [11]. These differences indicate that other mechanisms account for tumor progression and resistance to treatments, among which non-coding genome might be strongly involved.

Functional non-coding genome encompass DNA sequences that do not lead to protein coding products, which include ribosomal RNAs, transfer RNAs, micro RNAs, piwi-interacting RNAs and long non-coding RNAs [12]. Among them, long non-coding RNAs (lncRNAs) are key regulators of cellular processes, and are currently emerging as drivers for tumor aggressiveness and patients' outcome [13]. Herein we will provide insights of their oncogenic properties, discuss the interest of new classifications involving lncRNAs expression in clinical practice and introduce their contribution for optimal patient management.

## OVERVIEW OF LONG NON-CODING RNA LANDSCAPE

**Figure 1:**
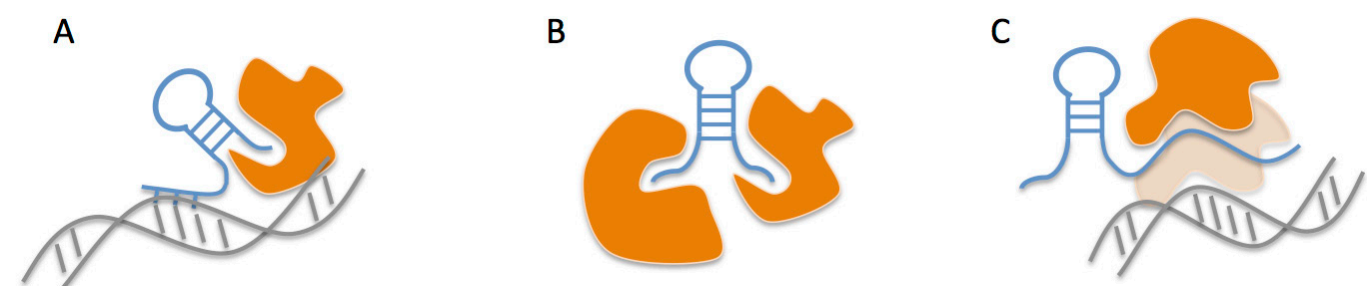
Main mechanisms of action of lncRNAs Adapted from Rinn et al. [16]. **A.** LncRNAs can guide proteins to DNA sequences, such as chromatin remodeling proteins. **B.** LncRNAs can act as scaffolds to induce the formation of functional protein complexes. **C.** LncRNAs can mimic DNA structures and act as decoys for proteins with DNA binding activity.

LncRNAs are eukaryotic RNAs > 200 nucleotides that do not have protein-coding capacity. Their expression is distinct from protein-coding genes and depends on the tissue and cellular context [14]. Most lncRNAs are intergenic or antisense transcripts that share transcription similarities with messenger RNAs. For instance, they lack an extended open reading frame and their transcription is dependent on the RNA polymerase II and are under the control of the transcriptional activators of the SWI/SNF complex. Finally, most of of the transcripts are capped and polyadenylated [15]. LncRNAs can be present in each cell compartment, where they interact with proteins and chromatin thanks to secondary structures such as stem loops and hairpins, acquired through post-transcriptional modifications. These secondary structures allow them to perform various functions. LncRNAs can act as scaffolds to bring protein complexes together, can guide proteins such as transcription factors to their DNA targets, bend chromatin to act as transcriptional enhancers, or act as decoys to wash out proteins from chromatin (Figure 1) [16]. These functions allow lncRNAs to have a crucial role on various cellular processes [17].

**Figure 2:**
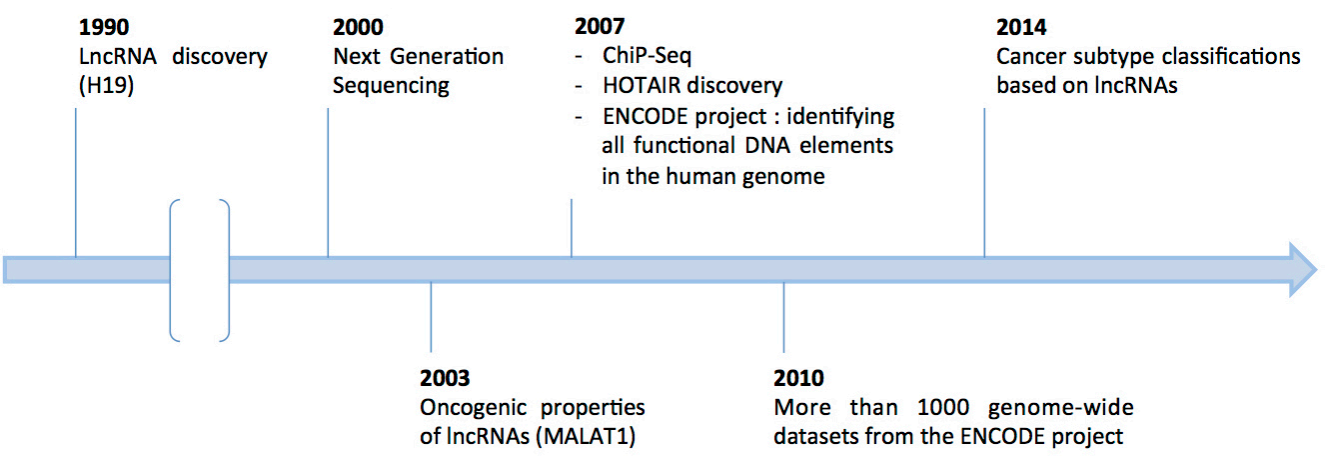
Major advances for lncRNA studies over time

LncRNA comprehension has been greatly enhanced by whole human genome analyses that provided a comprehensive dataset of human lncRNAs, detailing their expression, function, and distribution in the human genome (Figure 2) [18]. Still, the classification of lncRNAs remains to be unified, as they can be sorted according to various features: their structure, their sequence, their function, their metabolism, and their interaction with protein-coding genes or other known DNA elements [19].

Recent studies have highlighted the implication of lncRNAs in cancer progression, mainly through epigenetic regulation, activation of oncogenic pathways and crosstalks with other RNA subtypes [20,19]. Consequently, lncRNAs seem to be natural candidates as novel cancer biomarkers.

## ONCOGENIC PROCESSES ASSOCIATED WITH LNCRNA ALTERATIONS

### Epigenetic modifications

LncRNAs are able to interact with chromatin remodeling complexes, leading to modifications in the expression of target genes in *cis*, on the vicinity of the lncRNA sequence, or in *trans*, at independent loci throughout the genome [21]. HOTAIR was one of the first oncogenic lncRNA reported to be involved in cancer progression through genome-wide epigenetic reprogramming [22, 23, 24], through the interaction with polycomb repressive complex 2 (PRC2) subunits, a key chromatin remodeling complex involved in gene silencing [25]. In a cancer setting, HOTAIR recruits PRC2 subunits in promoter regions of tumor suppressor genes, which leads to their transcriptional repression and favours tumor progression. Accordingly, numerous lncRNAs are involved in regional or genome wide chromatin state regulation, such as MALAT-1 or ANRIL, among the most frequently expressed lncRNA in tumors [26, 27].

### Oncogenic signaling pathways

LncRNAs have a crucial role in the activation of oncogenic signaling pathways that drive the cancer phenotype [28]. The oncoproteins *MYC* and p53, frequently deregulated in cancers, are under the influence of lncRNA regulations. *MYC* gene alterations consist mostly in amplifications, as a result of copy gains of 8p24. It has been reported by Tseng et al [29] that lncRNA PVT1 is needed for MYC dependent oncogenesis. PVT1 is indeed localized in the same locus, and coamplified with MYC in 98% of *MYC* amplified tumors. Other lncRNAs are involved in the regulation of *MYC* expression, such as lncRNA XLOC_010588, downregulated in bladder cancer [30]. Conversely, transcriptional activation of oncogenic lncRNAs are directly mediated by MYC, such as CCAT-1 in colon cancer [31] and H19 in gastric cancer [32]. Regarding p53, it has been reported to be regulated by numerous non-coding RNAs [33]. Notably, lincRNA-p21, a downstream target of p53, links to ribonucleoprotein hnRNP-k to induce global p53 target gene repression and inhibition of apoptosis in lung cancer models [34]. MALAT-1 is also reported to inhibit *p53* gene expression and its target genes in various tumors [33]. In contrast, other lncRNAs increase apoptosis and are thus thought to be tumor suppressors, notably GAS5 in prostate cancer [35] and breast cancer cell-lines [36].

Among key oncogenic drivers, PI3K/MTOR/AKT and MAP kinases pathways are frequently promoted by lncRNAs alterations. ANRIL in gastric cancer represses miRNAs directed against mTOR [37]. LncRNA RMEL3 in necessary for the activation of BRAF and AKT in melanoma cell lines [38]. LncRNA PTENpg1 represses PTEN expression by recruiting DNMT3A and EZH2 to PTEN promoter region [39]. In contrast, PTENP1 acts as a decoy for regulators miRNAs that leads to increased PTEN function [40]. Similarly, KRAS1P also acts as a decoy for negative regulators of KRAS, thus enhancing KRAS function [40]. Other deregulations in proliferation pathways include the NFKB pathway, activated by BANCR in gastric cancer [41], and the lipid signaling molecules sphingosine kinases, activated by HULC in hepatocellular carcinoma [42].

Some lncRNAs are strongly associated with epithelial-mesenchymal transition (EMT) and cell migration. LncRNA-ATB in hepatocellular carcinoma [43] and MALAT-1 in bladder cancer [44] are positive regulators of TGF-β signaling, a key pathway for of EMT. Oncogenic lncRNAs GAPLINC in gastric cancer [45] and MVIH in non small cell lung cancer [46] upregulate CD44 and MMP2/9, proteins involved in cell migration. HOTAIR up-regulation is also associated with EMT, inducing downregulation of E-cadherin and high expression of Vimentin and Metalloproteinase 9 (MMP9) in colon cancer [47]. Conversely, BANCR acts as an anti-oncogene in non small cell lung cancer [48], as it impairs cell invasion through upregulation of E-cadherin, and downregulation of Vimentin and N-cadherin.

Other pathways notably regulated by lncRNAs include hormone signaling, cell cycle progression, or reactivation of development genes. In prostate cancer, NEAT1 [49] and PRNCR1 [50] are key elements of estrogen and androgen signaling, respectively. LncRNAs MIR31HG [51] and ANRIL are associated with cell cycle progression through the regulation of cyclin-dependent kinases inhibitors [52]. LncRNAs such as HOTTIP in hepatocellular carcinoma and TUG1 in lung cancer interact with *HOX* development genes, namely *HOXA13* and *HOXB7* [53, 54], involved in numerous oncogenic processes, such as proliferation, motility and angiogenesis.

The importance of lncRNAs in oncogenic mechanisms is such that various new implications will undoubtedly be unveiled in the years to come. The association of driver oncogenic alterations with lncRNA deregulations opens the way to integrative classifications using coding and non-coding genome.

### LncRNA and anticancer treatments

The importance of lncRNAs in the management of anticancer treatment is supported by their participation in drug resistance in cancer. In prostate cancer, numerous lncRNAs are associated with resistance to hormonal therapy: the oncogenic lncRNA NEAT1 is a transcriptional activator downtream of estrogen receptor α (ERα) and thus independent from androgen signaling [49], while lncRNAs PRNCR1 and PCGEM1 interact with and activate truncated androgen receptors, leading to androgen independent activation of androgen receptors [50]. In bladder cancer, the lncRNA UCA-1 induce resistance to cisplatin-based chemotherapy by upregulating the Wnt/β-catenin pathway [55]. In ovarian cancer, expression of HOTAIR, evaluated in 309 patients, is reported to be associated with resistance to carboplatin-based regimens, of unknown mechanism [56].

Conversely, expression of some lncRNAs might be predictors of response to anticancer treatments. This is the case with CCAT-1, a superenhancer of MYC in colon cancer, which transcription is highly sensitive to bromodomain inhibitors *in vitro* [57].Thus, it becomes clear that future biomarker studies will have to focus on lncRNAs to tailor individual treatments and to identify and overcome therapeutic resistance.

### Crosstalks with the RNA machinery

**Figure 3:**
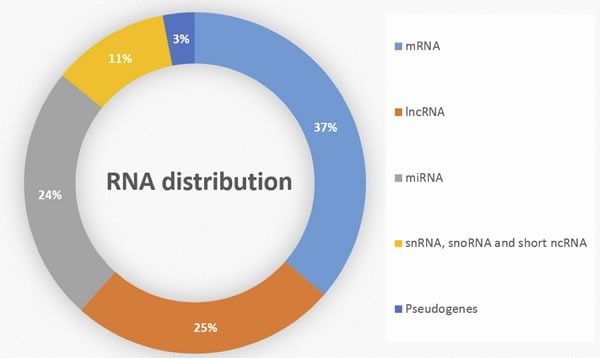
Distribution of RNA subtypes in human genome Adapted from Atianand et al. [60]. lncRNAs: long non-coding RNAs, miRNAs: micro RNAs, short ncRNAs: short non-coding RNAs, snRNAs: small nuclear RNAs, snoRNAs: small nucleolar RNAs.

LncRNAs interactions include crosstalks with other RNA subtypes, such as messenger and micro-RNAs, creating a network of interactions that can be involved in cancer mechanisms. Notably, lncRNAs are involved in miRNA regulation: the lncRNA ANRIL interacts negatively with the micro-RNAs miR99A and miR449A to promote mTOR and E2F1 expression [37]. Conversely, miRNAs can regulate lncRNAs activity, such as miRNA141, which suppresses HOTAIR expression [58]. Interactions between RNA subtypes have been notably explored in esophageal squamous cell carcinomas, where it has been reported that defined sets of lncRNAs, miRNAs and mRNAs encompass similar oncogenic features, such as apoptosis inhibition, cell cycle activation, proliferation, invasion and metastasis [59]. This suggests that diverse RNA subtypes work in cooperation for the acquisition of cancer properties. As lncRNAs encompass 25% of total RNAs in the human cell (Figure 3), their interaction network must be further studied to better grasp the full role of RNA crosstalks in pathology [60].

These data underscore the dramatic importance of lncRNAs in cancer. Interactions between lncRNAs and major oncogenic pathways support the role of lncRNAs in early steps of cancer development and their interest for more accurate cancer classifications. In addition, the unveiling of novel crosstalks between lncRNA and micro-RNAs raises the importance of epigenetic regulations in cancer, an expanding field of study.

## LNCRNAS PROFILING INTEGRATION IN CANCER CLASSIFICATIONS

### LncRNAs as prognostic biomarkers

As a result of the activation of oncogenic processes, lncRNA deregulations are associated with a more aggressive phenotype and drug resistance in cancer [13]. One of the first evidence of lncRNA involvement in cancer progression has been reported in breast cancer, where HOTAIR expression was being systematically increased compared to normal tissue. High expression of HOTAIR was associated with increased vascular invasion, advanced tumor stage, metastasis and poor prognosis [22]. In addition to breast cancer, overexpression of HOTAIR has been reported to be a marker for poor prognosis in various cancer types [61], including gastro-intestinal cancers [62, 63, 64, 65], urologic cancers [66, 67], gynecologic cancers [68, 69, 70], lung cancers [71, 72] and undifferentiated carcinoma of nasopharyngeal type [73]. So far, numerous lncRNA have been associated with poor prognosis in subsets of tumors, such as SCHLAP1 in prostate cancer [74], HOTTIP in hepatocellular carcinoma [53], gastric cancer [75] and colon cancer [76], or FAL1 in ovarian cancer [77]. Other are associated with good prognosis, such as NBAT-1 in neuroblastoma [78] or NKILA in breast cancer [79]. The most striking evidence of their prognostic impact come from a large-scale pan-cancer analysis conducted on 15 cancer types, which reported that 32 to 310 lncRNAs in each tumor had prognostic relevance [80].

Thus, lncRNAs might be considered as putative relevant prognostic markers for the clinic. As such, high expression of the lncRNA ENSG00000261582 is an independent marker of poor overall survival in lung and ovarian cancer in multivariate analysis [81]. Other robust studies indicate that high expression of HOTAIR in estrogen receptor negative breast cancer independently associated with lower overall survival in multivariate analysis [22, 82]. These observations provide the proof of concept that lncRNAs might be potent and discriminative prognostic markers, which can bring independent information from genomic, proteomic or clinicopathological features.

Thus, it seems crucial to take into account lncRNAs profiling to improve current cancer classifications, and conduct integrative analyses with known clinical, pathological and molecular features of cancers [83, 84].

### Integrative classifications

Du et al. report the first study assessing the role of lncRNAs in subtype classifications of 1,300 tissue samples among 4 different subtypes of cancer: ovarian, prostatic, lung SCC and glioma [81]. In this study, microarrays analyses have been performed to determine the lncRNAs expression profiles, with probes covering more than 10,000 lncRNAs identified through Ensembl and RefSeq sequence databases spanning the entire genome. LncRNAs were shown to display a specific expression pattern that correlated with established genomic classification in ovarian cancer, glioma, and lung squamous cell carcinoma. In ovarian cancer, unsupervised lncRNAs clustering identifed four groups of tumors matching with immunoreactive, mesenchymal, proliferative, and differentiated genomic profiles. In glioma, four groups were correlated with the classical, neural, proneural and mesenchymal profiles. In lung squamous cell carcinoma, lncRNA expression matched with basal, primitive, classical, and secretory-type gene expression profiling. The association between coding genome and lncRNAs points the relevance of lncRNA profiling to establish cancer subtypes classification. As lncRNA profiles can be established using RNAseq data that tend to be increasingly accessible, we believe that lncRNA profiling will be of great importance in the coming future.

Already, several reports explored the impact of lncRNA in cancer subtype classifications in different localizations including glioma, colon, breast, renal and endometrial cancers (Table 1). In glioma, the most frequent subtype of primitive brain tumors, consensus clustering of lncRNAs using 425 cases revealed three molecular subtypes: lncR1, lncR2 and lncR3 [85]. LncR1 subtype was associated with astroglial gene signature, epidermal growth factor (EGFR) amplification and patients displayed poor overall survival. On the contrary, lncR3 subtype correlated with oligodendritic gene signature, *IDH1* mutation, 1p/19q chromosomal deletions and patients had good prognosis. Finally, lncR2 represented an intermediary subtype regarding prognostic, with neuronal gene signature. Interestingly, this lncRNA classification correlates with various molecular prognostic factors, but it is still unknown whether lncRNA alterations are bound to specific genetic mutations such as IDH1, or chromatin remodeling genes alterations.

**Table 1:** Cancer subtype classifications based on lncRNA expression profiles

Cancer type	LncRNA database and selected set	Assessment of lncRNA expression	N	LncRNA classification	Molecular alterations	Prognosis
**Glioma**	**Refseq, Ensembl** 1979 lncRNAs with relevant expression in glioma	Microarrays	284	lncR1 lncR2 lncR3	Astroglial signature, EGFR amplification Neuronal signature, no recurrent alteration Oligodendroglial signature, IDH1 mutation, 1p/19q loss	**Overall survival** Poor Intermediate Good
**Colon**	**GEO** 6 lncRNAs associated with prognosis in colon cancer	Microarrays	895	High risk Low risk	N/A N/A	**DFS (150-months DFS)** Poor (51,49%) Good (78,09%)
**Breast**	**GENCODE** 1623 lncRNAs with relevant expression in breast cancer	RNA-sequencing	656	C1 C2 C3 C4	Basal-like HER2-enriched Luminal A Luminal A + luminal B	**Overall survival** Good Poor Good Poor
**Renal cell carcinoma**	**GENCODE** 1934 lncRNAs with relevant expression in renal cell carcinoma	RNA-sequencing	475	C1 C2 C3 C4	PBRM1 mutations, 9p deletion, BAP 1 mutations PBRM1 mutations Misclassified	**Overall survival (median)** Good (7,64 years) Poor (3,33 years) Good (not reached) Good (not reached)
**Endometrioid endometrial carcinoma**	**GENCODE** 1931 lncRNAs with relevant expression in endometrioid endometrial carcinoma	RNA-sequencing	191	Basal-like Luminal-like CTNNB1-enriched	MSI, POLE, p53 mutations, chromatin remodeling genes (EZH2, MLL) mutations ESR1, ERBB2 expression CTNNB1 mutations, PTEN loss	**No distinct prognostic groups**

GEO: Gene Expression Omnibus. DFS: disease free survival. EMT: epithelial mesenchymal trnasition. MSI: microsatellite instability, POLE: polymerase epsilon, N/A: not available

In colon cancer, a prognostic signature for disease-free survival has been established from six lncRNA which expression was strongly correlated with prognosis. This signature has been validated in 459 patients using the Gene Expression Omnibus (GEO) database [86]. This signature divides the population into two subgroups: high- and low-risk, according to lncRNA expression. However, stratification using the combination of both clinical staging (stages I/II *v* stages III/IV) and lncRNA signature (high-risk *v* low-risk) was capable to subdivide the patients into 4 groups with significantly distinct disease-free survival. This indicates that patients' stratification might be improved using integrative analysis of lncRNAs and clinical data. This study does not specify the presence of associated alterations, such as a deficient mismatch repair (dMMR) or a methylator phenotype (CIMP). Considering that lncRNAs act as broad epigenetic regulators, exploration of interactions between CIMP and lncRNA should be of great interest for the next future of colon cancer management.

In invasive breast cancer, we recently reported the lncRNAs portrait of 658 tumors and compared the data with the genomic PAM50 classification [87]. PAM50 is a molecular classification that identified four breast cancer subgroups, based on transcription profiling: basal-like, HER-2 enriched, luminal A, and luminal B. As PAM50 classification, we identified four clusters of lncRNAs clusters of lncRNAs have been identified. Those clusters correlated with PAM50 classification. Of note, while the three lncRNA clusters C1, C2 and C3 associated with basal-like, HER-2 enriched and luminal A groups and were highly correlated with their relative PAM50 mRNA classification, cluster C4 was not. Indeed, although C4 lncRNA cluster was associated with luminal signature and estrogen receptor (ER) expression, C4 cluster was not clearly capable of differentiating luminal A and luminal B subgroups. Thus, future studies are needed to explore the links between lncRNAs and estrogen receptors in breast cancers, as it is the case for H19 and HOTAIR [88, 89]. In addition, the relevance of lncRNA for prognostic classifications in breast cancer must be confronted to validated genomic classifications for the prediction of recurrence and prognosis in use in clinical routine [90].

Another cancer where lncRNA is important in defining tissue-specificity is clear-cell renal cell carcinoma (ccRCC). In 2010, ccRCCs have been classified in two molecular subgroups, type A and B, with distinct disease-free survival [91]. Effort form the TCGA working group helped identify four distinct subgroups with different outcomes, based on mRNA and miRNA expression [92]. More recently, using unsupervised lncRNA clustering of 475 primary tumor samples from the Cancer Genome Atlas, we reported four distinct lncRNA subgroups of ccRCCs associated with distinct prognosis, pathological features and molecular alterations, including specific alterations of chromatin-remodeling genes [93]. The prognostic value of lncRNA profiling was found to be independent from pathological grade and TNM stage. Cluster 2 was enriched for tumors harboring mutations in the chromatin-remodeling gene *BAP1*, and was strongly associated with high tumor grade and poor prognosis. In contrast, two clusters with better prognosis displayed *PBRM1* mutations, a component of *SWI/SNF* transcriptional activator. This suggests that ccRCCs subtypes have different epigenetic landscape alterations, which are closely linked with their lncRNA expression profile. However, how those lncRNAs are interacting with *PBRM1 and BAP1* remains to be explored. Of note, one subgroup was composed of misclassified tumours (chromophobe RCC and translocation RCC), suggesting the importance of lncRNAs in defining cell ontogeny.

Recent work focused on endometrioid endometrial carcinoma, the most frequent subtype of endometrial adenocarcinoma and usually associated with good prognosis. Unsupervised clustering of 1,931 lncRNAs significantly expressed in endometrioid endometrial carcinoma identified 3 subgroups: basal-like, luminal-like and CTNNB1-enriched [94]. Basal-like and luminal-like subgroups shared similarities for lncRNAs expression compared to their equivalent breast cancer subtype. The luminal subgroup was associated with expression of progesterone (PGR) and estrogen receptor (ESR1) genes, while the *CTNNB1* subgroup involved mutations in the β-catenin gene and PTEN loss. There was a trend towards poorer survival in the basal-like subgroup, which included more aggressive tumors that were enriched for p53 mutations and mutations of the *MLL* genes family. The interplay between lncRNA subtyping and alterations of chromatin remodeling genes might be an interesting area for the development of innovative therapies in this indication.

These studies suggest that lncRNA expression profile is correlated with genomic, genetic, pathological and clinical features of diverse neoplasms, but also brings independent prognostic value in various subsets of tumors. As such, lncRNAs are likely to be integrated in cancer classifications during the next decades.

## PERSPECTIVES

LncRNAs represent a new class of cancer cell regulators involved in diverse mechanism of oncogenesis, with new implications unveiled continuously. In the largest study to date, transcriptomic analysis of 91,013 expressed human genes in more than 7,256 tissue samples have shown that lncRNA may represent nearly 70% of human transcripts [95]. This number is considerably higher than expected, as lncRNA represented only 25% of the transcriptome in other series. This indicates that lncRNAs might outnumber coding RNAs by a large margin, which suggests that their importance in human biology is underestimated. In addition, precise understanding of the mechanisms involved in genome-wide epigenetic regulation is not fully fleshed out. Common patterns might be responsible for epigenetic regulation associated with different RNA systems [96]. To better understand these associations, integrative analysis of non-coding RNAs localizations and chromatin-RNA interactions across the tumor genome are needed. Further investigations are also underway to better understand the networks involving both coding and non-coding RNA machinery, regulating cellular processes [97]. International efforts are being made with the creation of participative RNA databases and computer-based simulations for molecular interactions, such as RNAbase [98], lncRNATor [99], and Cupid [100] projects, that will help further encompass the complexity and the promising potential of lncRNAs.

LncRNAs are relevant and potent biomarkers for cancer subtype analyses and prognostic stratifications with tissue-specific expression. Their implementation in clinical practice is around the corner, pending validation studies to assess the reproducibility of those classifications and the input of their integration in patient's management. Development of therapies targeting lncRNAs will be a major challenge. Previous attempts at RNA targeting faced difficulties for treatment distribution in the tumor site, which remain to be resolved [101]. The concurrent development of biomarkers will be of great interest in the fields of diagnosis, prognosis assessment, and prediction to identify the population eligible to future treatments targeting lncRNAs. New insights might be provided through the emergence of liquid biopsies. Indeed, HOTAIR, HULC and H19 expression levels in plasma have been demonstrated to correlate with outcome in series of patients with metastatic colon cancer [102], hepatocellular carcinoma [103] and gastric cancer [104], respectively. This is of high interest considering the possibilities of using lncRNAs as potent circulating biomarkers.

As lncRNA profiling represents an exciting tool in cancer management, its implementation will be a key player in the integration of molecular medicine into clinical practice and personalized care.
